# Led, unled, and unacknowledged: care staff’s experiences of registered nurses’ leadership close to older adults

**DOI:** 10.1186/s12913-026-15286-4

**Published:** 2026-08-06

**Authors:** Therese Stien, Lise-Lotte Jonasson, Karin Josefsson

**Affiliations:** 1 Faculty for Nursing and Health Science, Nord University, Bodø, 8026 Norway; 2 Department of Nursing Science, School of Health and Welfare, Jönköping University, Jönköping, Sweden

**Keywords:** Older adults, Care staff, Registered nurse, Home healthcare, Leadership, Group interview

## Abstract

**Background:**

Older adults in municipal home healthcare receive allocated care, including personal hygiene, nutritional aid, medication, and wound care. Care staff in this sector are a diverse group, comprising both educated and uneducated personnel. Registered nurses (RNs) lead the care close to older adults. Leadership is recognized as influencing a group towards a common goal and affects patient safety and the quality of care. However, little is known about how care staff experience RNs’ leadership close to older adults.

**Methods:**

This study is explorative, with an inductive design. Three group interviews with care staff (*n* = 13) were conducted in three municipalities in Norway from June to October 2025. The group size was four to five care staff. Data were analysed with qualitative content analysis.

**Findings:**

The findings gave insights into care staff’s experiences of RNs’ leadership close to older adults. The experiences were categorized into three categories: led, unled, and unacknowledged, and 11 subcategories. Leadership was experienced as person-dependent, necessary, providing supportive quality assurance, entailing collaboration, and showing trust, as well as absent, unclear, and self-absorbed. RNs could underestimate care staff’s competence, overlook them or fail to notice their uncertainty in their leadership. The findings were that care staff’s experiences of RNs’ leadership close to older adults were diverse and nuanced, encompassing both supportive and conflicting aspects.

**Conclusions:**

RNs’ leadership close to older adults could benefit from being clearly defined, which could reduce the person-dependent aspects of this leadership. In structural changes such as task shifting, leadership should be explicit. This study suggests that decision makers in home healthcare should allocate resources to ensure an organizational structure that provides RNs with the prerequisites to lead, to prevent care staff from experiencing being unled and unacknowledged.

**Supplementary Information:**

The online version contains supplementary material available at https://doi.org/10.1186/s12913-026-15286-4.

## Background

The Norwegian home healthcare (HHC) service is publicly funded and organized by municipalities [1]. Municipal HHC serves the entire population and includes several healthcare services; however, the majority of people receiving nursing care at home are older adults [2]. Despite older adults’ diverse ageing trajectories [3], multimorbidity affects older adults as its prevalence increases with age [4, 5]. Older adults in HHC have a high degree of frailty; 76% are classified as moderately or highly frail [6]. HHC is provided so that people can achieve better coping and function, increase years of life and improve their quality of life [7]. Older adults in HHC receive allocated care, for example help with personal hygiene, wound care, nutritional aid, and medication.

In municipal HHC, registered nurses (RNs) and care staff with and without formal education make up most of the workforce. Care staff can be a diverse group, comprising of educated personnel such as certified nursing assistants (CNAs), individuals currently in training, and care workers without formal education. According to national legislation, all care staff in HHC are health workers [8]. “Certified nursing assistant”, on the other hand, is a protected title with a certification [7]. Beyond their basic education, CNAs can also have additional training such as a higher vocational diploma.

The HHC sector has a high rate of absence due to sick-leave and vacant positions, and is the sector with the highest level of long-term sick leave in Norway [9]. Like other countries, Norway is experiencing difficulty in recruiting and retaining educated health workers, and municipalities are facing challenges in adequately meeting the population’s health care needs [10]. The number of care staff without healthcare education is increasing and absent RNs could be replaced by uneducated workers [11].

Moreover, HHC is a growing sector. The proportion of older adults in Norway is projected to double between 2021 and 2075 [12]. At the same time, the number of individuals of working age is decreasing [13], resulting in pressure on healthcare systems to provide care for more people. In the last decades HHC has become more advanced and people with complex needs are receiving care and treatment in their own homes, in line with political governance [14]. Resources and competence requirements in the municipal healthcare services have not increased in step with the increase in needs [11]. Care staff in HHC provide direct patient care, including nursing services, medical treatment, and practical assistance with daily living. Task shifting, the process of matching skills to needs and opportunities (often shifting certain tasks to less specialized personnel), is seen as having possible contributions in mitigating increased needs with fewer specialized personnel [15].

Nursing practice in HHC can be characterized in three patterns: to be vigilant, to be an all-rounder and to act with independence [16]. When assessing risk in HHC, health care professionals experience professionalism as adapting the care to here-and-now situations. Moreover, doing so in teams is essential for delivery of high quality of care [17]. Quality of care for older people in HHC includes, and is built on, relationships between the healthcare workers and the older person, continuity of care, and tailoring of the care to the individual older person’s needs. As such, quality of care has organizational and structural aspects, and can be understood as a measurable construct [18]. Quality of care and patient safety are both affected by leadership [19, 20].

Leadership is recognized as influencing a group towards a common goal [21]. In HHC, RNs lead the care close to older adults [22]. Previous research has described RNs’ leadership close to older adults as a complex role, with RNs required to be “multi-artists”; the role involves trust and control, application of skills, mutual relationships and awareness of the individual’s needs and wholeness [22]. Nursing leadership can be described as having clear objectives, collaborating with others to solve problems, and recognizing interpersonal interactions and communication skills as essential [23]. RNs have the capacity to influence the health outcomes of patients, the effectiveness of their teams and the functioning of the health system [24]. They plan, evaluate and customize the care [25, 26]. For this, they need both organizational skills and clinical knowledge and they are through their education qualified to lead [27].

Leadership is identified as a key responsibility of all RNs, not only those in formal leadership positions [28]. Those working in HHC view their leadership as involving negotiating accountability for nursing activities, gaining an overview of nursing activities and managing these within a limited time [29]. RNs’ leadership can be considered person-oriented [30, 31], meaning that they lead by using themselves as persons in relationships with other people. RNs have been reported to perceive their ability to lead close to older adults as high [32]. Student nurses are educated in leadership and practice models have been shown to improve leadership [33].

Older adults in HHC experience RNs’ leadership close to them as RNs being “their own RN”, implying a caring and personal relationship alongside showing professional competence and performing nursing interventions [34].

In this study, RNs’ leadership is defined as leading close to older adults, their next of kin and the care staff; management is not included. In HHC, care staff close to older adults are led by RNs. However, little is known about how care staff experience RNs’ leadership. Therefore, it is essential to explore their experience of RNs’ leadership close to older adults in HHC.

### Aim

The aim is to explore how care staff experience registered nurses’ leadership close to older adults in municipal home healthcare.

## Method

### Design

An explorative and inductive design was utilized [35]. A convenience sample was used. Three group interviews with care staff (*n* = 13) were conducted in three municipalities in Norway from June to October 2025. The group size was four to five care staff. Data were analysed with qualitative content analysis [36–38].

### Participants and procedure

Inclusion criteria for group interviews were care staff, with or without education, who had worked close to older adults in HHC for one year or more [35]. Managers in municipalities were first contacted by telephone, whereafter they received an invitation e-mail with an information letter, which was distributed to workers within the inclusion criteria. One municipality used a contact person, not manager, for handling the invitation. In two municipalities, the interviewer presented the study in advance. One municipality organized the group before the interviewer met with care staff. Contact persons at HHC arranged a time and a private room for the interview. An overview of care staff characteristics is shown in Table 1.

**Table 1:** Care staff’s characteristics (*n* = 13)

Characteristics		
Age	min–max (mean)	26–62 (43)
Female	*n* (%)	11 (85)
Work experience in municipal home healthcare, years	min–max (mean)	1–34 (11)
Certified nursing assistants	*n* (%)	12 (92)
Care staff with training entailing a higher vocational diploma	*n* (%)	4 (30)
Care staff working in a rural area	*n* (%)	9 (69)
Care staff working in an urban area	*n* (%)	4 (31)

### Data generation

An interview guide was discussed and developed (see Supplementary file) by the research team during several meetings [39, 40]. It consisted of three main questions: (1) What do you think when I say registered nurse leadership close to older adults in municipal home healthcare? (2) How do you experience being led by registered nurse leading close to older adults in municipal home healthcare? (3) Before ending the interview, is there anything you wish to share about your experience of being led by registered nurses close to older adults in municipal home healthcare? Follow-up questions were, for example, In what way? Can you elaborate? Is there anything else you want to add or say regarding this? Interviews were audio-recorded and lasted for an average of 52 (range 50–56) minutes. Interviews were transcribed on the same day to prevent loss of information.

### Analysis

Due to focus on subject, contexts and variation the interviews were analysed following qualitative content analysis according to Graneheim, Lundman and Lindgren [36–38]. The analysis followed an inductive approach, meaning categories emerged through the analysis. The transcribed interviews were read through several times and units of analysis were divided into meaning units by identifying variations and similarities. Analysis was mainly manifest; however, the data also facilitated some latent analysis. The meaning units were then condensed, keeping the core while shortening the text. The condensed meaning units were abstracted and labelled with a code. The context was considered when labelling the condensed meaning unit with a code. Based on similarities and differences, codes sharing commonality were sorted into subcategories. When subcategories were found to be internally homogeneous and externally heterogeneous, they were sorted into categories with consistent threads. The analysis was discussed on several occasions and at several levels within the research team. Table 2 shows an example of the data analysis process.

**Table 2:** Example of the data analysis process

Meaning unit	Condensed meaning unit	Code	Subcategory	Category
… I’ve had a case that involved several RNs who were very professionally strong, and I found it valuable to discuss issues with them. You receive guidance – on how things should or could have been done differently …	I had a case involving several highly skilled RNs, and it was valuable to discuss issues with them and get guidance on how things should/could have been done differently.	Professional guidance	RNs’ leadership provides supportive quality assurance	Led

### Preunderstanding

The researchers’ preunderstanding was discussed in several meetings before, during, and after data generation and analysis. The first author (TS) was the interviewer, an RN with previous experience of working in HHC. A handwritten reflexive journal was utilized by the interviewer during data generation and analysis to avoid preunderstanding shaping the understanding of the empirical data and leading the analysis [41]. The analysis steps were discussed in depth within the research team in several meetings intended to prevent preunderstanding from overshadowing the empirical data and to promote critical reflection on how researchers’ perspectives influenced the analytical process. This was done to ensure that the researchers’ preunderstanding was not taking precedence over data.

### Ethical considerations

This study adheres to good research practice by following the National Research Ethics guidelines as outlined by the National Committee for Research Ethics in the Social Sciences and the Humanities [42]. The project was assessed by the Regional Committee for Medical Research Ethics Northern Norway, REK North (D. No.: 753491), and ethics approval was deemed unnecessary, in accordance with national legislation. The Helsinki Declaration was originally developed for medical research [43]; however, by adapting the underlying principles, such as informed consent, protections of participants and scientific integrity, this study follows good scientific practice. All participants were given the option not to participate. There was no dependence between the participants and the researchers. The participants’ identification and ensured privacy were protected in line with the guidelines provided by the Norwegian Agency for Shared Services in Education and Research (SIKT) (registered D. No.: 478281).

### Findings

The study explored how care staff experienced RNs’ leadership close to older adults in municipal HCC. The analysis shows these experiences organized into three categories: led, unled, and unacknowledged. Altogether the categories entailed 11 subcategories (Table 3).

**Table 3:** Care staff’s experience of registered nurses’ (RNs’) leadership close to older adults in home healthcare

Subcategories	Categories
RNs’ leadership is person-dependent RNs’ leadership is necessary RNs’ leadership provides supportive quality assurance RNs’ leadership entails collaboration RNs show trust in their leadership	Led
RNs’ leadership is absent RNs’ leadership is unclear RNs’ leadership is self-absorbed	Unled
RNs do not notice our uncertainty RNs underestimate our competence RNs overlook us	Unacknowledged

### Led

Being led entailed experiencing RNs’ leadership close to older adults as person-dependent, meaning that care staff experienced leadership as dependent on which RN was on duty. The overall experience of RNs’ leadership was varied and dependent on the competency, the experience and especially the personality of the RN in charge that day. When care staff viewed RNs as dedicated to the care of older adults, they experienced them as leading with clarity. Care staff expressed confidence in the guidance and goals set by someone they perceived as dedicated. They linked personality traits such as extroversion to clear leadership.

… he knows what he’s doing, and he loves talking about it [the care]. I always trust his evaluations … (Participant 4)

RNs’ leadership was experienced as necessary. The care staff expressed the necessity for someone to organize care for older adults and coordinate efforts within and outside of municipal HHC. The care staff placed the responsibility for these efforts on RNs. RNs’ leadership was also experienced as providing supportive quality assurance, for example when care staff were evaluating care, training for new procedures, or handling unclear situations. The care staff described leadership and guidance as a quality check during training for new procedures. They stated that RNs guided them in handling challenging situations with older adults and experienced relying on RNs to decisively resolve challenges during workdays.

… in home healthcare, we have no back-up. We face many sad and unpleasant situations, and when we need help, we rely on the registered nurse to tell us whether it’s acceptable or not … (Participant 6)

The care staff experienced that RNs’ leadership entailed collaboration between them and the RNs. There was an experience of RNs’ leadership as based on interprofessional relationships. When care staff shared their observations about the older adults with the RNs, the RNs would ask questions and might also seek the care staff’s opinion on how to proceed. Collaboration with RNs was viewed as a vital part of a functional workday and was related to how care staff experienced RNs’ leadership.

… the most important thing is collaboration, which is based on trust and mutual respect. That’s the core of everything, both when they delegate and when we approach them … (Participant 6)

As with collaboration, the care staff experienced RNs’ leadership as showing trust in the care staff. The care staff felt included and seen when RNs took their evaluations and observations seriously. They described instances when they were dismissed, but they also described an overall sense of being trusted by the RNs. Trust was seen as a sign that RNs respected the competence of and evaluations made by care staff, and trust was described as important for care staff to be open towards RNs.

… it creates a sense of trust when they ask about a patient they don’t know well, because they can’t make assessments without knowing what we observe in the field … (Participant 12)

### Unled

RNs’ leadership close to older adults in HHC was also an experience of not being adequately led. This experience of being unled was based on absent, unclear or self-absorbed leadership.

The experience of RNs’ leadership as absent occurred when care staff did not receive the guidance they needed or when they viewed RNs as reluctant to make decisions, leaving care staff with a “wait and see” experience. The care staff also described that the RNs were absent from the care, and that they only saw them in the office. This resulted in care staff experiencing RNs as unable to make assessments and lead close to older adults.

… I was performing a wound dressing, and there was disagreement about how we should do it. I assumed that she, as the registered nurse, would tell us. She said nothing, and we just stood there. I put something on, but I have no idea whether it was right or wrong, because I didn’t get any response from her … (Participant 4)

RNs leadership was experienced as unclear when it was difficult to understand the reasoning behind decisions made by an RN, or when RNs’ decisions were vague. The care staff then had concerns about the RNs’ competence in care of older adults and expressed questioning the RNs’ evaluations and guidance. Care staff also made a point of stating that they were able to fill the leadership role of RNs. Some care staff expressed vagueness in how RNs took responsibility and led the care of older adults. In these situations they experienced the RNs’ leadership as unclear.

… she rarely seems to know what’s going on. She just sits there and delegates, without grasping the situation. I asked her a question: ‘This patient needs this and that.’ ‘Yes, that’s fine,’ she said. And I find myself thinking that she has never actually been out there [providing care] … (Participant 11)

The care staff also experienced some RNs’ leadership as self-absorbed, benefiting the RNs themselves and their older adults (i.e. older adults closest to the RN). This entailed care staff experiencing that RNs prioritized themselves and ensured an easy workday for themselves. The care staff also experienced RNs prioritized the older adults closest to them. Care staff felt that their own work and the older adults in their care were being deprioritized, and that some RNs did not value care staff as equal colleagues. They reported situations in which they asked for help and the RNs did not offer guidance or help, saying that they would not perform such work.

… it has to do with the registered nurse’s prioritization of patients … the ones they look after are their own. The ones who must wait are ours. They are lightening their workload while we are lagging behind because our problems are not being addressed … (Participant 9)

### Unacknowledged

The findings indicates that care staff experienced RNs’ leadership as RNs not seeing them, by RNs not noticing their uncertainty, underestimating their competencies, and overlooking them in their leadership. For example, RNs did not notice care staff’s uncertainty when handling new medications or performing new procedures, and care staff were told to “just do it”. The care staff pointed out that to be overlooked by the RN in their leadership resulted in them having to raise their voices, during reports or during delegation, in an effort to be heard. They expressed having to stand up for themselves and their workday. Care staff trying to convey information or needs of older adults were sometimes not taken seriously until the RNs came to see for themselves. This felt like prior observations and evaluations by care staff were dismissed and their competence was underestimated.

… a person in need of care required a lift, and we tried to communicate that two people were needed. Not before the registered nurse saw for themselves did we get that second person … (Participant 5)

… I’ve learned that I need to speak up louder. It often happens that things are left for later, and after a while, ‘later’ starts to pile up quite a lot … (Participant 10)

## Discussion

The aim of this study was to explore how care staff experience RNs’ leadership close to older adults in municipal HHC. The findings indicates that care staff’s experiences of RNs’ leadership close to older adults were diverse and nuanced, encompassing both supportive and conflicting aspects. Findings indicates that care staff experienced being unacknowledged in RNs’ leadership by the RNs perhaps underestimating their competence. This highlights that, when supervising and delegating in multi-tiered teams, RNs must have an understanding of each team member’s skill level and competencies [44].

On the other hand, the care staff also experienced being shown trust in RNs’ leadership. Trust is a central component in leadership and cooperation [45]. These conflicting findings could reflect RNs’ perceptions of care staff’s skill level and competencies, combined with a neutral level of trust. Previous research indicates RNs have neither a high nor a low degree of trust in care staff [32, 46]. It can also be pointed out that this may be the result of organizational pressure, if RNs show trust in care staff while at the same time underestimating their competence.

The findings indicate care staff experiencing themselves as unled, and they expressed being able to fill RNs’ leadership role, indicating either a lack of leadership or an underestimation of or failure to experience RNs’ leadership. This might be linked to both task shifting and RNs’ invisible work. Invisible work can be perceived as simple routines when in reality it involves comprehensive problem solving [47]. If RNs’ leadership is invisible work to care staff, it can be underestimated and lead to the experience of being unled or unacknowledged. On the other hand, visible work is recognized in organizations [47]. However, organizing and task shifting based on visible work could obscure the comprehensive nature of HHC work. RNs’ leadership close to older adults demands that the RNs perform like multi-artists, as reported by Claesson et al. [22]. When the complexity of the work is invisible to care staff, this can lead to experiences of being unled, as this study reflects. Stakeholders such as RNs themselves, care staff and managers may also lack the language, insight or confidence in the RNs’ contribution [47]. Clinical leadership has been recognized as an important factor for implementing changes in HHC [48]. However, the extent of invisible work in the healthcare sector has been shown to impact patient safety [49]. If changes in organizational structures are based solely on visible work, unforeseen challenges could arise [47]. Together with previous research, this study indicates a need for the RNs’ responsibility and leadership role close to older adults to be clarified, made visible, and discussed at higher levels of decision making [29, 50], perhaps especially in the context of task shifting and organizational changes.

Paradoxically to care staff experiencing being unled, this study shows that RNs’ leadership close to older adults was also experienced as a necessity. Leadership is the process of influencing a group towards common goals [21]. Care staff experienced that RNs guided them in challenging situations and initiated appropriate actions when necessary. RNs in HHC are perceived to tie up loose ends [51], and to be leading meaning makers among the HHC staff [29]. Being able to act independently has been identified as one of the key characteristics of and requirements in nursing practice in HHC [16]. In fact, RNs leading close to older adults in HHC share similarities with clinical leaders as described by Stanley et al. [52], and it has been reported that leaders without formal leadership positions can be “a hidden treasure” in the clinical setting [53]. At the same time, experiencing a fellowship with co-workers is also a vital part of work satisfaction in HHC [54]. This indicates a need for both leadership and fellowship between care staff and RNs in HHC, adding to the complexity of the RNs’ leadership role in HHC.

The care staff in this study experienced some RNs as prioritizing themselves and their own tasks over those of the care staff. In these situations, the care staff experienced not being led by the RNs. HHC is constantly changing and the traditional role of Norwegian RNs as close bedside practitioners is being challenged by new organizational structures being implemented across municipalities [55–57]. Care staff in this study described safeguarding their workday (i.e. against excessive workload) as well as for older adults’ needs under RNs’ leadership close to older adults. RNs handle the most complex patients and work tasks in HHC [58]. By prioritizing care for older adults with complex needs, they might give care staff the impression that other adults could be receiving lower-quality care. As a result, the care staff feels they must advocate for their patients. The reasons for prioritizing some patients and tasks over others may not be conveyed to care staff, or due to RNs’ workload. Moreover, RNs coordinate care in HHC [26], and spend over twice as much of their worktime on administrative tasks as other staff in HHC [58]. Most of their worktime is spent in the indirect care for patients, which raises the need for this work to be made explicit [59]. The amount of indirect care work could be contributing to care staff’s experience of being unled. Organizational structures may not allocate enough time for RNs to handle care staff’s concerns, with care staff experiencing being unacknowledged as a result. It should also be pointed out that especially newly qualified RNs may not yet have developed the leadership skills needed [60]. By ensuring adequate training for care staff to handle work in indirect care situations, such as administrative work tasks, care staff could become more independent from RNs. A transformational leadership style from managers has been found to indirectly contribute to hindering adverse events through RNs in direct care positions clinical leadership [61]. By developing the leadership skills of RNs in direct care positions, decision makers could contribute to hindering adverse events.

### Limitations

This study had a sample size of *n =* 13, including two male participants. However, this represents the gender distribution of care staff in most municipal HHC in Norway. Care staff could be a diverse group; in this study 12 out of 13 care staff had completed healthcare education, despite education in healthcare not being an inclusion criterion. The findings may primarily reflect the experiences of healthcare-educated personnel. The transferability of the findings should therefore be considered in light of the sample characteristics. Using managers as intermediaries could have affected recruitment.

The interviews and analyses were performed in Norwegian and then translated into English. The interviewer is an RN with previous work experience in HHC. There were no prior connections between participants and interviewer. However, the interviewer’s background was known to participants. The interviewer experienced care staff as open and talking without restraint. The findings may be transferable to contexts beyond care for older adults in HHC, albeit with different considerations for different care services. It is important to emphasize that the RNs’ experiences were not the focus of this study.

## Conclusion

This study reveals care staff’s experience of RNs’ leadership close to older adults as one of being led, unled, and unacknowledged. RNs’ leadership close to older adults could benefit from being clearer defined, which could reduce the person-dependent aspects of this leadership. In structural changes such as task shifting, RNs’ leadership close to older adults should be explicit. This study suggests that decision makers in home healthcare should allocate resources to ensure an organizational structure that provides RNs with the prerequisites to lead, to prevent care staff from experiencing being unled and unacknowledged.

### Implications for practice

This study strongly indicates the need to make RNs’ leadership close to older adults visible and explicit, as well as to better educate RNs in leadership skills and competencies. The findings indicate that the participating care staff tended to place some responsibilities for their workday on RNs. This could be addressed by ensuring that responsibility is appropriately allocated to the organizations and decision makers who formally hold it. The study also indicates that RNs’ leadership close to older adults could benefit from clearer organizational guidelines to support their leadership.

## Supplementary Information

Below is the link to the electronic supplementary material.

Supplementary Material 1

## Data Availability

The data are available from the corresponding author upon reasonable request.
