# Supplementary material for: Led, unled, and unacknowledged: care staff’s experiences of registered nurses’ leadership close to older adults
Source: BMC Health Serv Res. 2026 Aug 6;26:1522. doi: 10.1186/s12913-026-15286-4 (PMC13732914; doi:10.1186/s12913-026-15286-4)
Supplement: Supplementary file 1 [file 12913_2026_15286_MOESM1_ESM.docx]

# Interview guide

## Introduction

The participants are welcomed and thanked for their participation. Orally repeat the information related to the aim of the study: to explore registered nurses’ leadership close to older adults in municipal home healthcare, defined as leading close to the older adults, where next of kins and care staff also are.

Address participants confidentiality and the use of background information, such as age and gender to describe the group of participants as a whole. Estimated interview duration of up to one hour. Confirm consent, which was signed before and submitted to the interviewer. Clarify whether there are any questions.

## Main questions

1. Can you tell me what you think about registered nurses’ leadership close to older adults in municipal home health care?
2. Can you tell me about your experiences of registered nurses’ leadership close to older adults in municipal home healthcare?

**Follow-up questions**

Can you tell me more?

Can you describe it?

What do you mean by …?

Can you give some examples of that?

Can you elaborate?

**Closing question**

Is there anything you would like to share regarding your experience of registered nurses’ leadership close to older adults in municipal home healthcare, before we conclude?
